# ASIC-E4: Interplay of Beta-Amyloid, Synaptic Density and Neuroinflammation in Cognitively Normal Volunteers With Three Levels of Genetic Risk for Late-Onset Alzheimer's Disease – Study Protocol and Baseline Characteristics

**DOI:** 10.3389/fneur.2022.826423

**Published:** 2022-02-09

**Authors:** Anniina Snellman, Laura L. Ekblad, Mikko Koivumäki, Noora Lindgrén, Jouni Tuisku, Merja Perälä, Lila Kallio, Riina Lehtonen, Virva Saunavaara, Jani Saunavaara, Vesa Oikonen, Richard Aarnio, Eliisa Löyttyniemi, Riitta Parkkola, Mira Karrasch, Henrik Zetterberg, Kaj Blennow, Juha O. Rinne

**Affiliations:** ^1^Turku PET Centre, University of Turku and Turku University Hospital, Turku, Finland; ^2^Department of Psychiatry and Neurochemistry, Institute of Neuroscience and Physiology, The Sahlgrenska Academy at the University of Gothenburg, Mölndal, Sweden; ^3^Auria Biobank, Turku University Hospital, University of Turku, Turku, Finland; ^4^Department of Medical Physics, Turku University Hospital, Turku, Finland; ^5^Department of Biostatistics, University of Turku, Turku, Finland; ^6^Department of Radiology, Turku University Hospital, University of Turku, Turku, Finland; ^7^Department of Psychology, Åbo Akademi University, Turku, Finland; ^8^Clinical Neurochemistry Laboratory, Sahlgrenska University Hospital, Mölndal, Sweden; ^9^Department of Neurodegenerative Disease, UCL Institute of Neurology, London, United Kingdom; ^10^UK Dementia Research Institute at UCL, London, United Kingdom; ^11^Hong Kong Center for Neurodegenerative Diseases, Hong Kong, China

**Keywords:** Alzheimer's disease, *APOE*, preclinical, biomarker, beta-amyloid, TSPO (18 kDa translocator protein), SV2A ligand, neuroinflammation

## Abstract

**Background:**

Detailed characterization of early pathophysiological changes in preclinical Alzheimer's disease (AD) is necessary to enable development of correctly targeted and timed disease-modifying treatments. ASIC-E4 study (“Beta-Amyloid, Synaptic loss, Inflammation and Cognition in healthy *APOE* ε4 carriers”) combines state-of-the-art neuroimaging and fluid-based biomarker measurements to study the early interplay of three key pathological features of AD, i.e., beta-amyloid (Aβ) deposition, neuroinflammation and synaptic dysfunction and loss in cognitively normal volunteers with three different levels of genetic (*APOE-*related) risk for late-onset AD.

**Objective:**

Here, our objective is to describe the study design, used protocols and baseline demographics of the ASIC-E4 study.

**Methods/Design:**

ASIC-E4 is a prospective observational multimodal imaging study performed in Turku PET Centre in collaboration with University of Gothenburg. Cognitively normal 60–75-year-old-individuals with known *APOE* ε4/ε4 genotype were recruited via local Auria Biobank (Turku, Finland). Recruitment of the project has been completed in July 2020 and 63 individuals were enrolled to three study groups (Group 1: *APOE* ε4/ε4, *N* = 19; Group 2: *APOE* ε4/ε3, *N* = 22; Group 3: *APOE* ε3/ε3, *N* = 22). At baseline, all participants will undergo positron emission tomography imaging with tracers targeted against Aβ deposition (^11^C-PIB), activated glia (^11^C-PK11195) and synaptic vesicle glycoprotein 2A (^11^C-UCB-J), two brain magnetic resonance imaging scans, and extensive cognitive testing. In addition, blood samples are collected for various laboratory measurements and blood biomarker analysis and cerebrospinal fluid samples are collected from a subset of participants based on additional voluntary informed consent. To evaluate the predictive value of the early neuroimaging findings, neuropsychological evaluation and blood biomarker measurements will be repeated after a 4-year follow-up period.

**Discussion:**

Results of the ASIC-E4 project will bridge the gap related to limited knowledge of the synaptic and inflammatory changes and their association with each other and Aβ in “at-risk” individuals. Thorough *in vivo* characterization of the biomarker profiles in this population will produce valuable information for diagnostic purposes and future drug development, where the field has already started to look beyond Aβ.

## Introduction

Over fifty million people worldwide live with dementia, and the number is expected to rise to 78 million by 2030 (1). Alzheimer's disease (AD) causes over two thirds of the dementia cases (2). To cope with this challenge, AD drug development has shifted its focus toward disease-modifying treatments and targeting the underlying pathophysiological events as early as possible, even before the onset of clinical symptoms (3, 4). Recent approval of Aducanumab by the U.S. Food and Drug Administration as the first disease-modifying treatment for AD (https://www.fda.gov/news-events/press-announcements/fda-grants-accelerated-approval-alzheimers-drug) gives hope for new drugs targeting various aspects of AD pathophysiology to become available in the future. However, this development also creates an increasing need for suitable biomarkers to better characterize the different biological stages of AD and substitute clinical endpoints in future therapeutic trials with varying targets.

Biologically, AD is characterized by extracellular beta-amyloid (Aβ) plaques and intracellular tau aggregates in the brain, however, complexity behind the disease process is increasingly recognized (5). Alterations in Aβ metabolism are traditionally presented to be the earliest changes, leading to further hyperphosphorylation and aggregation of tau into intraneuronal neurofibrillary tangles, but the relationship between the two pathologies is now known to be more complex and synergistic (6). In addition, neuroinflammation, characterized by activation of the immune cells in the central nervous system (CNS), and synaptic dysfunction and loss are known to be present throughout the AD pathophysiological process (7). These alterations in the brain begin to develop decades before clinical symptoms arise, and the course of AD has been re-evaluated to be a progressive continuum from a preclinical “silent” phase to a clinically manifested dementia phase, characterized by the early presence of various biological markers of the ongoing disease process (8, 9). Thus, the earliest brain changes and their associations with each other can only be investigated in pre-symptomatic individuals, who are at increased risk of AD in the future.

The strongest genetic risk factor for sporadic AD is the ε4 allele of the apolipoprotein E gene [*APOE*, (10)]. *APOE* is polymorphic, and from the three different alleles (*APOE* ε2, *APOE* ε3 and *APOE* ε4), one ε4 allele increases the lifetime risk of AD 3–4 fold, and two ε4 alleles 9–15 fold (11). In the CNS, Apolipoprotein E is produced mainly by astrocytes and microglia, and it functions as a lipid transporter delivering cholesterol and other lipids to neurons, thus maintaining and restoring membranes and synaptic integrity (12). In AD pathogenesis, *APOE* ε4 is involved with both gain of toxic functions, such as increased Aβ aggregation, tangle formation and brain atrophy, as well as loss of neuroprotective functions, such as reduced synaptic and vascular function (13). Especially early Aβ deposition has repeatedly been shown to be present in the brain of *APOE* ε4 carriers (14, 15) already prior to any cognitive changes (16). Even though the mechanisms are still not understood in detail, *APOE* ε4 most likely contributes to increased amyloid aggregation by deficient clearance of Aβ from the brain compared to ε3 and ε2 (17). In addition to Aβ and tau pathology, neuroinflammation has been suggested to be one link between *APOE* mediated increased risk for AD; Since *APOE* is mainly expressed in astrocytes and microglia, the cells responsible for important immune functions in the CNS, *APOE* ε4 is likely to have also a direct effect on glial functions, independent of Aβ or tau (17). Throughout this article we describe individuals with *APOE* ε4ε4, *APOE* ε4ε3 and *APOE* ε3ε3 genotype as having high, intermediate and low risk for sporadic AD, respectively. However, it is important to note that this description only highlights the differences in relative *APOE*-related risk associated with each genotype and is not used for describing the absolute risk of future AD of individuals with these genotypes.

Development of imaging biomarkers for AD-related pathophysiological change has provided the research community with a valuable tool for investigating the regional and temporal course of different brain changes in AD *in vivo*. Positron emission tomography (PET) with specific radioligands targeting Aβ deposition and tau aggregates are already well-established imaging biomarkers of AD. They are used for biological definition of the disease and for studying the progression and relationship between these core AD pathophysiological changes (9). In addition to the core pathologies, PET ligands targeting TSPO expressed in activated glial cells (18–20), and recently also synaptic vesicle glycoprotein 2A (SV2A) in presynaptic vesicles (21–24), provide a way for also investigating the neuroinflammatory and synaptic components of the disease process *in vivo*. Despite the obvious value of various imaging biomarkers for diagnostic and research purposes, the methods are often expensive, invasive, result in a radiation dose and have limited availability. Thus, more easily accessible blood biomarkers for AD have recently been under vigorous research and shown promise as early diagnostic markers [Reviewed in (25) and (26)]. Ultrasensitive immunoassays and immunoprecipitation combined with mass spectrometry have made it possible to measure various proteins characteristic for AD pathophysiology from blood, including different soluble Aβ species (27), soluble tau and phosphorylated tau species (28–32), neurodegeneration by plasma neurofilament light chain (NfL) (33), and gliosis by plasma glial fibrillary acidic protein (GFAP) (34–37). However, it is still important to validate the performance of these blood-based biomarkers in different clinical and research populations, against gold standard PET methods and in longitudinal studies across the AD continuum.

The aim of the ASIC-E4 (“Beta-**A**myloid, **S**ynaptic loss, **I**nflammation and **C**ognition in healthy *APOE* **ε4** carriers”) study is to uncover the early interplay of three key pathological features of AD, i.e., synaptic dysfunction and loss, neuroinflammation and Aβ deposition, in cognitively normal individuals with either high, intermediate, or typical *APOE*-related genetic risk of late-onset AD. By utilizing state-of-the art neuroimaging and fluid biomarker measurements, the ASIC-E4 study aims to clarify whether differences in regional neuroinflammation and synaptic density can be detected *in vivo* already in cognitively normal subjects differentiated only by their *APOE* genotype and how they associate with each other, Aβ deposition, cognitive performance, and fluid blood biomarker findings in different *APOE* genotypes. In addition, after a 4-year follow-up, our aim is to evaluate how the early neuroimaging findings associate with changes in cognitive performance and blood biomarker levels in this valuable at-risk population.

## Methods and Analysis

### Study Design

**Figure 1:**
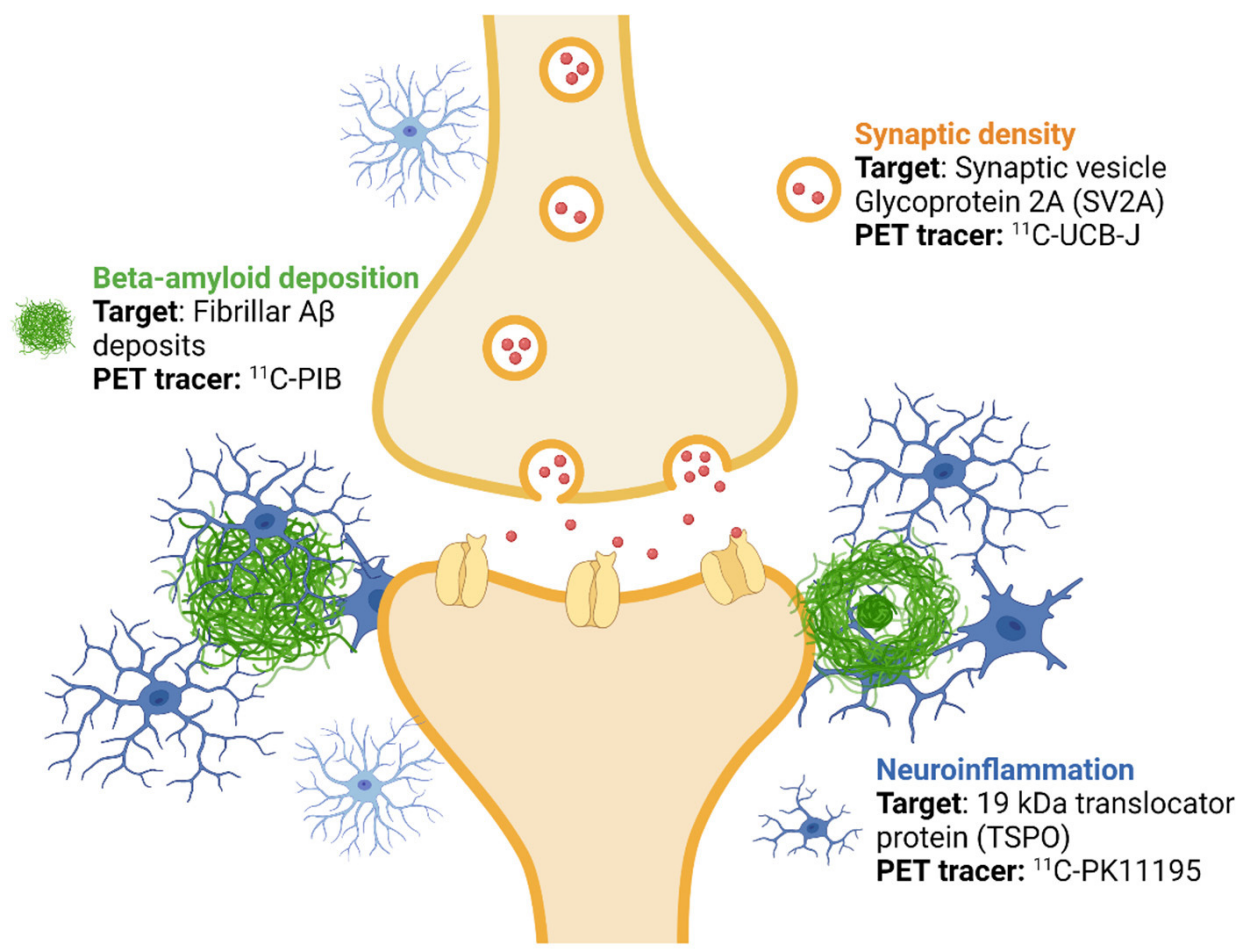
Imaging targets and used positron emission tomography (PET) tracers. ASIC-E4 is a prospective, observational multi-tracer imaging study, where three different positron emission tomography (PET) tracers are used to investigate beta-amyloid (Aβ) deposition, activation of glial cells and synaptic density in individuals with three levels of genetic (*APOE-*related) risk for sporadic Alzheimer's disease. The figure was created with BioRender.com.

ASIC-E4 is a prospective multimodal imaging study aiming to investigate the interplay of Aβ deposition, neuroinflammation and synaptic changes in cognitively normal individuals at different *APOE*-related genetic risk of AD (Figure 1). The study has been approved by Hospital District of South-West Finland (27.4.2018), and Scientific Advisory Boards of Turku PET Centre (26.3.2018) and Auria Biobank (30.1.2018). ASIC-E4 study is conducted following the guidelines of both National Advisory Board on Research Ethics in Finland, and the European Code of Conduct for Research Integrity by All European Academics. In addition, Declaration of Helsinki, Good Clinical Practice, and EU legislation and General Data Protection Regulation are followed. All used research material and data will be collected and analyzed during the project from participants recruited for the study. This study is observational, and does not include any studied interventions, thus it has not been prospectively registered to ClinicalTrials.gov.

### Power Calculation

We performed power calculations based on previously published data with the three used PET tracers, aiming for 90 % power (1-β = 0.9, α = 0.05) to detect 10–20 % differences in regional tracer binding between the cognitively normal *APOE* ε4/ε4 and *APOE* ε3/ε3 groups. Based on the calculations, with nine participants per group, we would be able to detect a 20 % difference in mean ^11^C-PIB standardized uptake value ratios (SUVRs) (1.2 vs. 1.5, standard deviation [SD] 0.2) (38); with 21 participants a 20 % difference in ^11^C-PK11195 binding potentials (0.37 vs. 0.44, SD 0.07) (18); and with 17 participants a 10 % difference in ^11^C-UCB-J binding potentials (3.0 vs. 2.7, SD 1.9) (21).

Due to the used multi-tracer approach, and thus higher risk that subjects will suspend their participation before completing all three PET scans, we aimed for recruiting 20–25 participants per group. The calculations were performed for differences between the homozygous *APOE* ε4/ε4 and *APOE* ε3/ε3 carriers, however, a gene dose effect is expected to be seen in heterozygous *APOE* ε4/ε3 carriers, even though it might not reach statistical significance.

### Objectives and Hypothesis

Our pre-defined objectives for the study are:

(i) To quantify differences in regional Aβ deposition (^11^C-PIB binding), neuroinflammation (^11^C-PK11195 binding), synaptic density (^11^C-UCB-J binding) and structural brain changes between *APOE* ε4/ε4 and *APOE* ε4/ε3 carriers with increased *APOE*-related genetic risk for sporadic AD, and homozygous *APOE* ε3/ε3 controls with typical *APOE*-related genetic risk for sporadic AD.
(ii) To study differences in blood biomarker levels between the three different *APOE* genotypes and cognitively normal amyloid positive and amyloid negative individuals.
(iii) To investigate regional and voxel-wise associations between brain Aβ deposition, neuroinflammation, and synaptic density with each other, with cognitive performance, with structural brain changes, and with blood biomarker levels both in the whole cohort and within the three different *APOE* genotypes.
(iv) To investigate the association between baseline neuroimaging findings and changes in cognitive performance and blood biomarker concentrations after 4-year follow-up.

Based on current knowledge and theoretical premise behind the research we hypothesize that:

(i) Cognitively normal *APOE* ε4/ε4 and *APOE* ε4/ε3 carriers show regionally increased ^11^C-PIB and ^11^C-PK11195 binding and decreased ^11^C-UCB-J binding compared to *APOE* ε3/ε3 carriers in brain regions typical for early tau and Aβ pathology. Risk allele dose effect is visible both in ^11^C-UCB-J (*APOE* ε4/ε4 < *APOE* ε4/ε3 < *APOE* ε3/ε3) and ^11^C-PIB and ^11^C-PK11195 uptake (*APOE* ε4/ε4 > *APOE* ε4/ε3 > *APOE* ε3/ε3).
(ii) Due to direct effect of toxic Aβ and tau oligomers on synapses, also ^11^C-PIB-negative *APOE* ε4 carriers with less fibrillar amyloid accumulation show decreased ^11^C-UCB-J binding in regions of early Aβ accumulation. Low synaptic density and high glial activation in *APOE* ε4 carriers are associated with lower baseline cognitive performance, and increased abnormality in blood biomarkers for AD pathology.
(iii) Increased abnormality in blood biomarker levels is present already in cognitive normal *APOE* ε4 carriers compared to *APOE* ε3/ε3 controls.
(iv) Abnormal baseline neuroimaging findings are associated with 4-year temporal changes in cognitive performance.

### Selection of Subjects

#### Inclusion and Exclusion Criteria

Predefined inclusion criteria for the ASIC-E4 study were:

(i) *APOE* ε4/ε4, *APOE* ε4/ε3 or *APOE* ε3/ε3 genotype.
(ii) 60–75-years of age.
(iii) Mini-Mental State Examination (MMSE) score ≥ 25 points.
(iv) Consortium to Establish a Registry for Alzheimer's Disease (CERAD) neuropsychological battery total score > 62 points (39).

The cut point for CERAD total score is defined as two standard deviations below the mean score of cognitively normal individuals in a previously published study on an European population (40). Participants are expected to be cognitively unimpaired as described in the syndromal staging of cognitive continuum in the AD research framework, i.e., cognitive performance of all participants was within expected range of that individual based on all available information (9).

Predefined main exclusion criteria for the ASIC-E4 study were:

(i) Dementia or cognitive impairment, including reported subjective memory complaints.
(ii) Previous diagnosis of other neurological or psychiatric diseases.
(iii) Previous diagnosis of diabetes.
(iv) Previous diagnosis of chronic inflammatory condition and related continuous use of anti-inflammatory medication
(v) Contraindication for magnetic resonance imaging (MRI) or PET (e.g., claustrophobia, presence of ferromagnetic objects in the body, previous high radiation doses).

#### Recruitment

Recruitment was done in collaboration with the local biobank (Auria Biobank, Turku, Finland, study number: AB17-2549). The biobank has access to blood samples from individuals who had previously signed a biobank consent, and an additional informed consent allowing the biobank to contact them if they are suitable for participating in a research study. *APOE* genotype had already been determined from a subset of the blood samples (APOE genotyping for methodological details), allowing the biobank to directly contact persons with either *APOE* ε4/ε4, *APOE* ε3/ε4 or *APOE* ε3/ε3 genotype. As only ~ 2–3% of the blood samples are expected to be homozygous *APOE* ε4/ε4, it would not have been feasible to recruit a group of *APOE* ε4/ε4 homozygotes without this valuable collaboration.

**Figure 2:**
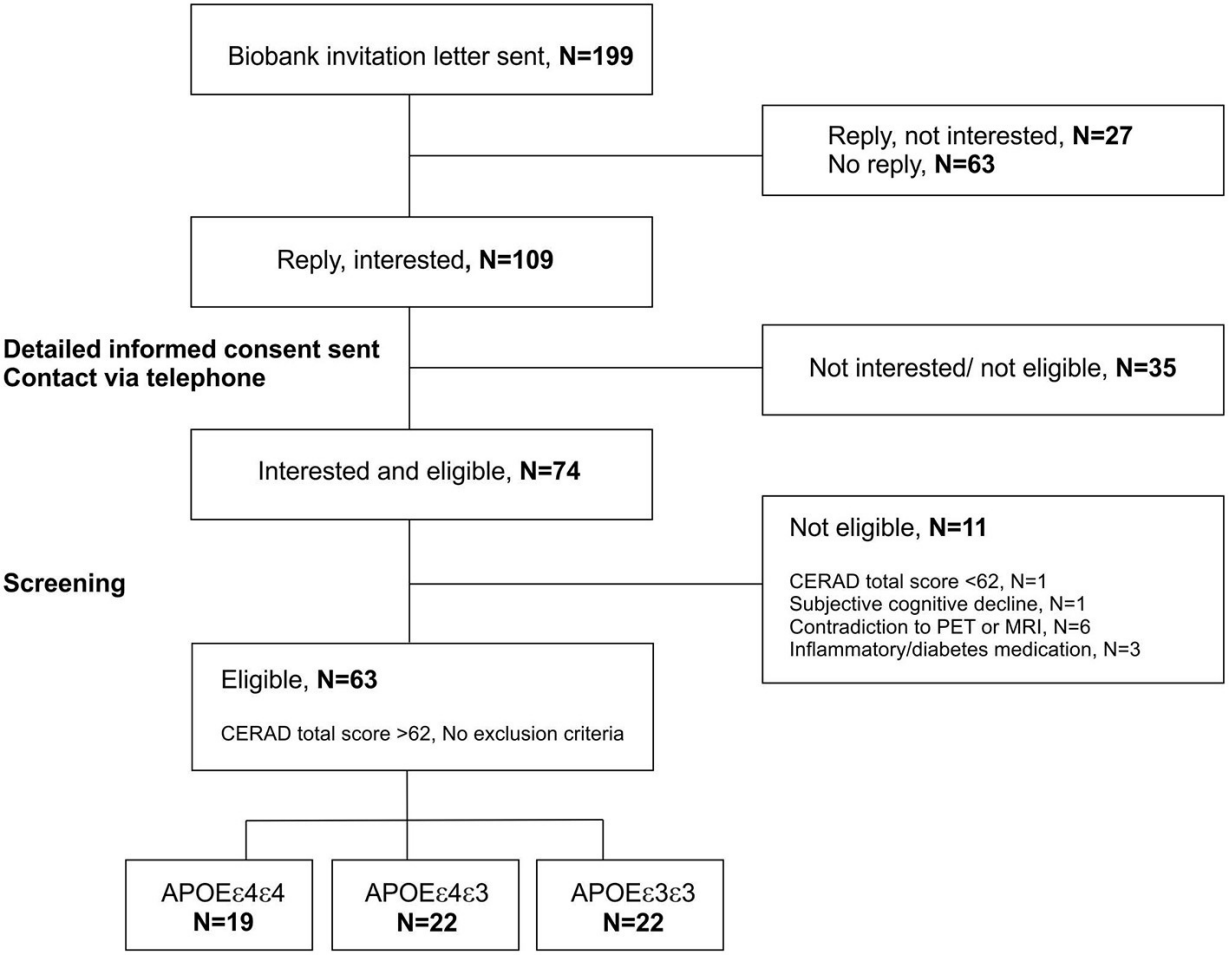
Recruitment scheme for the ASIC-E4 study. Recruitment is done in collaboration with the local biobank (Auria Biobank, Turku, Finland). The biobank was able to send invitation letters based on a biobank consent, directly to individuals with known *APOE* genotypes. The signed letters of interest including the contact information of the invited individuals, and written informed consent for transforming the *APOE* status from the biobank to the study site were returned to the responsible researcher by the invited individuals. Subsequently, researchers contacted interested individuals by telephone and if no obvious exclusion criteria was present, invited them to a screening visit. At screening, all interested individuals were interviewed and evaluated with Consortium to Establish a Registry for Alzheimer's Disease (CERAD) neuropsychological test battery. If calculated CERAD total score was >62 points, and no other exclusion criteria was present, individuals were enrolled to the study. APOE, apolipoprotein E gene; PET, positron emission tomography; MRI, magnetic resonance imaging.

Altogether 199 invitation letters were sent based on a biobank consent to people with either *APOE* ε3/ε3 (76 letters), *APOE* ε3/ε4 (66 letters) or *APOE* ε4/ε4 (57 letters) genotype during years 2018–2020 (Figure 2). Letters were sent in four batches balanced by age and gender, depending on the recruitment status after previous batches. From the individuals who received the invitation to participate, 109 returned a signed letter of interest and wanted to receive more information about the study. At this point, interested individuals also gave a written informed consent that the *APOE* status can be given from the biobank to the study site to ensure enrolment to different groups in a balanced way. Detailed study plan and informed consent were sent to all interested candidates, and everyone was subsequently contacted via telephone. During the conversation, the full study protocol was discussed, and if no clear exclusion criteria were found based on the telephone interview, and if the candidate was still interested in participating, they were invited to a screening visit to Turku PET Center.

#### Screening

During the screening visit, ASIC-E4 study protocol was discussed in detail and the informed consent was signed by all potential participants. Subsequently, more detailed medical history (e.g., current and past medications, previous medical diagnosis, past head traumas) and general health related personal information (education, smoking, family history for AD) were inquired. After the interview, CERAD neuropsychological battery was performed to all potential participants, to ensure that only cognitively unimpaired individuals were enrolled.

All medical information, CERAD total score and individual subtest scores collected during the screening visit were reviewed by a neurologist, who together with the person performing the screening based on this information made the final enrolment decision.

#### *APOE* Genotyping

*APOE* genotype data used for the ASIC-E4 study were obtained from Auria Biobank, Turku, Finland (Study number: AB17-2549). Genomic DNA was extracted from EDTA-anticoagulated whole blood samples from consented donors using the Chemagic DNA Blood Kit (CMG-1091, PerkinElmer) according to the manufacturer's instructions. *APOE* genotype analysis was performed at Turku University Hospital, Clinical Microbiology and Immunology Laboratory with a Taqman SNP genotyping assay (Applied Biosystems, ThermoFisher). The genotypes were determined using the TaqMan Genotyper Software (ThermoFisher).

### Study Protocol and Timeline

**Figure 3:**
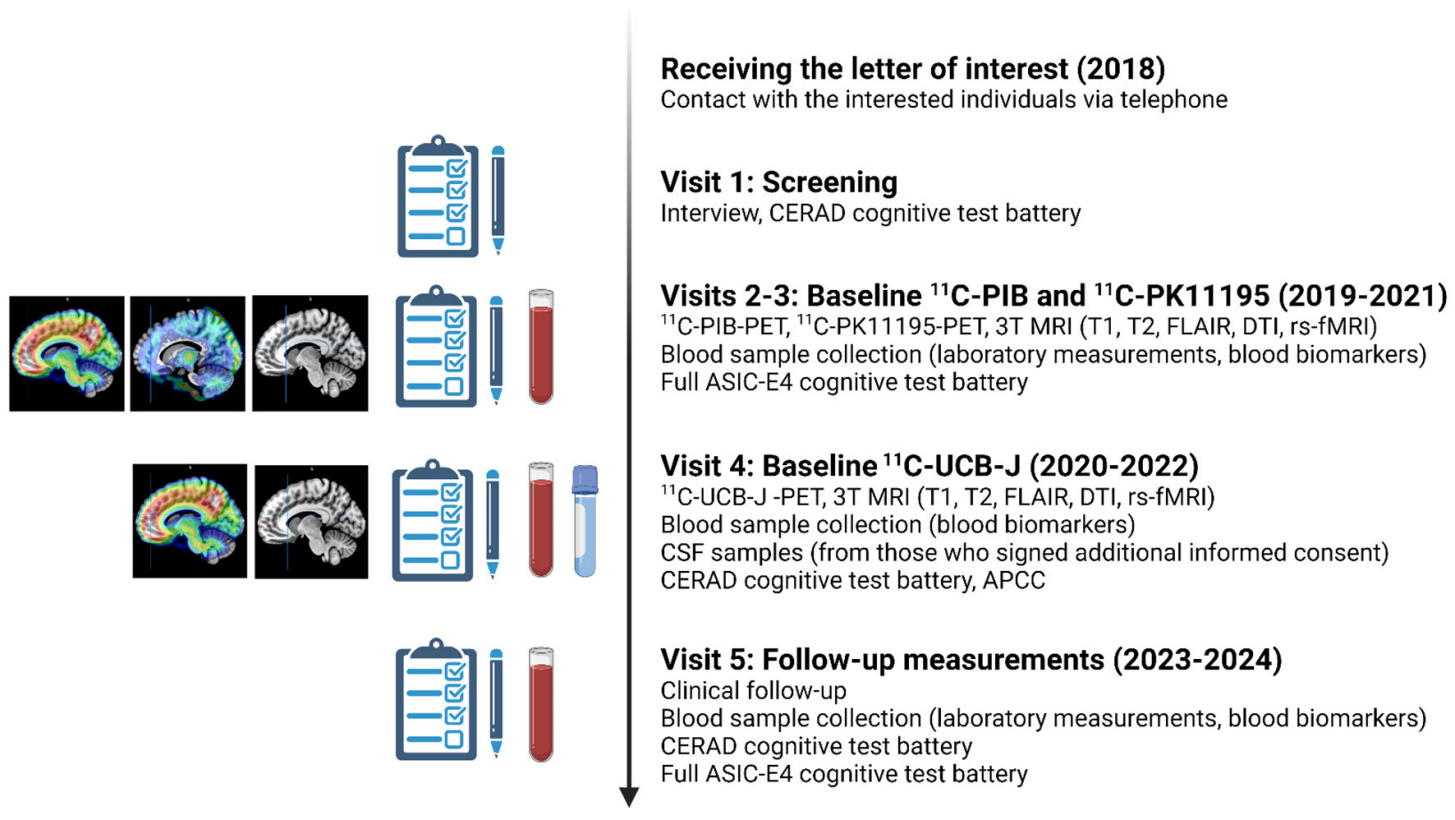
Planned study visits during the ASIC-E4 study. All included ASIC-E4 participants will visit Turku PET Center and Turku University Hospital 3–5 times during years 2019–2022. In addition, all will be invited to a follow-up visit approximately 4 years from screening (starting 2023). APCC, Alzheimer's Prevention Initiative Composite Cognitive Test Score; CERAD, Consortium to Establish a Registry for Alzheimer's Disease; CSF, cerebrospinal fluid; DTI, diffusion tensor imaging; rs-fMRI, resting state functional magnetic resonance imaging; FLAIR, fluid attenuated inversion recovery PET, positron emission tomography; MRI, magnetic resonance imaging. The figure was created with BioRender.com.

Detailed study protocol and planned visits to study site are illustrated in (Figure 3).

All enrolled participants will visit the PET Center 3–5 times for baseline PET imaging, MRI, blood sample collection and extensive neuropsychological evaluation. All measurements are aimed to be performed during a time interval as short as possible, preferably within a 3-month period. However, due to a delay in ^11^C-UCB-J availability, baseline ^11^C-UCB-J scans will be acquired approximately 12-18 months after baseline ^11^C-PK11195/^11^C-PIB/MRI for each participant. Due to this, an amendment to the study protocol was performed, and additional MRI, revised cognitive test battery and blood samples will be collected with the ^11^C-UCB-J scan to enable accurate analysis of the ^11^C-UCB-J PET data, and evaluation of association between ^11^C-UCB-J binding and blood biomarker levels at the same time point (see Figure 3 for details). It should be noted that the additional MRI scans and cognitive testing was not designed for follow-up purposes.

CSF samples are collected near ^11^C-UCB-J scans from those who give an additional informed consent for a lumbar puncture.

Four years after the initial visits (starting in January 2023), all participants will be invited for a follow-up visit. All participants will come to Turku PET Center for a clinical follow-up, interview and CERAD neuropsychological battery, similarly to the screening visit. New venous blood samples for laboratory measurements and blood biomarker analysis will be taken and stored for follow-up analysis. In addition to the CERAD test battery, all participants will go through another round of neuropsychological testing with the same ASIC-E4 test battery performed at baseline.

### Imaging

#### MRI

Non-contrast brain MRI is performed with a 3 Tesla MRI scanner for all study participants. Due to an unexpected change in the MR scanner availability at Turku PET Center during the data collecting period, imaging will be performed with two different devices. First batch of baseline scans are done using Philips Ingenuity 3.0 T TF PET-MR (Philips Healthcare, Amsterdam, the Netherlands), and the rest of the baseline scans will be performed with Philips Ingenia 3.0 T systems (Philips Healthcare, Amsterdam, the Netherlands). A 32-channel head coil will be used with Ingenuity PET-MR system and a 20-channel dS head coil with Ingenia system.

All participants will undergo MRI at two different time points; First MRI scan is done at proximity (± 3 months) to the baseline ^11^C-PK11195 and ^11^C-PIB PET scans (with either Philips Ingenuity and or Philips Ingenia). Due to the unexpected delay in ^11^C-UCB-J availability, another MRI with the exact same imaging protocol will be done at the time of ^11^C-UCB-J scan (all performed with Philips Ingenia) to ensure timely reference image for PET image analysis and volumetric data to be combined with ^11^C-UCB-J data.

MRI sequences included in the ASIC-E4 imaging protocol include 3D T1-weighted sequence, T2-weighted sequence, T2-weighted fluid-attenuated inversion recovery (FLAIR) sequence, resting state functional MRI (rs-fMRI) sequence, and diffusion tensor imaging (DTI) sequence. In addition, B0 Field Mapping and DTI TOPUP sequences will be acquired and their data will be utilized in analysis to minimize image distortions in rs-fMRI and DTI data. Used parameters and details of each sequence for both of the used MR scanners are listed in Tables 1, 2.

**Table 1:** Used MRI sequences using Philips Ingenuity 3.0 T TF PET-MR (Philips Healthcare, Amsterdam, the Netherlands).

**Sequence**	**Slice orientation**	**Parallel imaging factor**	**Nr of slices**	**Field of view (mm^**2**^)**	**Slice gap (mm)**	**Voxel size (mm^**3**^)**	**Repetition time (ms)**	**Echo time (ms)**	**Flip angle (**°**)**	**Bandwidth (Hz/Px)**	**Other relevant parameters of the sequence**
T1W 3D TFE	sagittal	SENSE 2	176	256 × 256	0	1 × 1 × 1	8.1	3.7	7	192	Shot interval: 3,000 ms IR delay: 1,075 ms
T2W TSE	transverse	SENSE 1.5	46	230 × 186	0.5	0.45 × 0.45 × 3.0	4,320	80	90	201	Refocusing control: On / 120 Turbo Factor: 15
3D VISTA	sagittal	SENSE 2 × 2	180	256 ×256	0	1 × 1 × 1	8,000	337	90	501	IR delay: 2,400 ms Fat Suppression: SPAIR
rs-fMRI EPI	transverse	SENSE 1.8	34	230 × 230	0	1.8 × 1.8 × 4.0	3,000	35	90	1,583	Number of scans: 200 Fat Suppression: SPIR
Field map (B0)	transverse	SENSE 2	80	256 × 256	0	2 × 2 × 2	1,120	10	18	2,337	Delta TE: 2.36 ms
DTI 64	transverse	SENSE 1.8	80	256 × 256	0	2 × 2 × 2	6,700	120	90	1,787	SPIR, b-value: 1,000 s/mm^2^, diffusion directions: 64
DTI TOPUP	transverse	SENSE 1.8	80	256 × 256	0	2 × 2 × 2	6,700	120	90	1,787	Phase encoding direction reversed compared to DTI

*DTI, Diffusion tensor imaging; Flair, Fluid-Attenuated Inversion Recovery; rs-fMRI, Resting-state Functional Magnetic Resonance Imaging; TFE, Turbo Field Echo; TSE, Turbo Spin Echo*.

**Table 2:** Used MRI sequences using Philips Ingenia 3.0 T systems (Philips Healthcare, Amsterdam, the Netherlands).

**Sequence**	**Slice orientation**	**Parallel imaging factor**	**Nr of slices**	**Field of view (mm^**2**^)**	**Slice gap (mm)**	**Voxel size (mm^**3**^)**	**Repetition time (ms)**	**Echo time (ms)**	**Flip angle (**°**)**	**Bandwidth (Hz/Px)**	**Other relevant parameters of the sequence**
T1W 3D TFE	sagittal	SENSE 2	176	256 × 256	0	1 × 1 × 1	8.1	3.7	7	181	Shot interval: 3,000 ms IR delay: 1,075 ms
T2W TSE	transverse	SENSE 1.3	40	230 × 181	1	0.45 × 0.45 × 3.0	4,438	80	90	168	Refocusing control: On / 120 Turbo Factor: 15
3D FLAIR	sagittal	SENSE 3 × 2	180	256 × 256	0	1 × 1 × 1	4,800	340	90	718	IR delay: 1,650 ms Fat Suppression: SPIR
rs-fMRI EPI	transverse	SENSE 1.8	34	230 × 230	0	1.8 × 1.8 × 4.0	3,000	35	90	1,403	Number of scans: 200 Fat Suppression: SPIR
Field map (B0)	transverse	SENSE 2	80	256 × 256	0	2 × 2 × 2	1,120	7	18	434	Delta TE: 2.36 ms
DTI 64	transverse	SENSE 1.8	80	256 × 256	0	2 × 2 × 2	6,700	120	90	1,468	SPIR, b-value: 1,000 s/mm^2^, diffusion directions: 64
DTI TOPUP	transverse	SENSE 1.8	80	256 × 256	0	2 × 2 × 2	6,700	120	90	1,468	Phase encoding direction reversed compared to DTI

*DTI, Diffusion tensor imaging; Flair, Fluid-Attenuated Inversion Recovery; rs-fMRI, Resting-state Functional Magnetic Resonance Imaging; TFE, Turbo Field Echo; TSE, Turbo Spin Echo*.

All MRI images are read by a neuroradiologist, for identification of any possible other pathologies in the brain.

#### PET Imaging

All PET scans will be performed with brain dedicated ECAT high-resolution research tomograph (HRRT, Siemens Medical Solutions, Knoxville, TN), with a spatial resolution of 2.5 mm. First, antecubital veins are cannulated to enable intravenous injection of the used radioligands. An individual thermoplastic mask is prepared and used to reduce head motion during the scans. Each participant is imaged with three different PET tracers specified below.

##### ^11^C-PIB-PET

The first PET scan is performed with ^11^C-PIB, a gold standard radioligand developed for imaging fibrillar Aβ aggregates (41). ^11^C-PIB is produced in-house with a previously published protocol (42). After intravenous administration of the tracer (dose aimed at 500 MBq, minimum 250 MBq) participants are advised to wait 30 min sitting or lying down, before they are moved into the scanner. Collection of the emission data is initiated at 40 min post injection and the scan duration will be 50 min. PET scan is followed by a 6 min transmission scan using a ^137^Cs point source for attenuation correction. List-mode data is histogrammed into 6 × 5 + 2 × 10 min time frames, and reconstructed with 3D ordinary Poisson ordered subset expectation maximization algorithm (OP-OSEM3D) with 16 subsets and 8 iterations and a voxel size of 1.22 × 1.22 × 1.22 mm.

##### ^11^C-PK11195-PET

The second PET scan performed to evaluate the early differences in regional neuroinflammation is performed using ^11^C-(R)-PK11195, a radioligand targeting the 18 kDa translocator protein (TSPO) known to be expressed in the mitochondrial membrane of activated glial cells (43). ^11^C-PK11195 is produced in-house with previously published method (44). A transmission scan is acquired first, and 60 min dynamic emission data collection will be started simultaneously with intravenous injection of the tracer (dose aimed at 500 MBq, minimum 250 MBq). List mode data is histogrammed into 17 timeframes (2 × 15; 3 × 30; 3 × 60; 7 × 300; 2 × 600 s) and reconstructed using the same method as with ^11^C-PIB imaging. No blood samples are collected during the scans.

##### ^11^C-UCB-J-PET

The third PET scan utilizes ^11^C-UCB-J, a tracer targeting synaptic vesicle protein SV2A. First scans (*N* < 10) will be performed with arterial blood sampling, allowing for full kinetic modeling of the data and subsequent comparison of the data to simplified methods used for the remaining scans. Arterial blood samples are collected manually every 15 s for the first 3 min, and thereafter at 4, 6, 8, 10, 15, 20, 25, 30, 45, 60, and 90 min post injection. Additional samples for analyzing radioactive metabolites and unchanged tracer fraction are collected at 3, 8, 15, 30, 45, 60, and 90 min post injection. After evaluating the first scans, protocol will be simplified, and arterial blood samples will no longer be collected. After intravenous tracer injection (dose aimed at 500 MBq, minimum 250 MBq), a 90 min dynamic emission scan is performed, followed by a 5-min transmission scan. List mode data is histogrammed into 29 timeframes (6 x 30, 7 x 60, 16 x 300 s) and reconstructed using OP-OSEM3D algorithm with 10 subsets and eight iterations and with point spread function modeling to improve further the spatial resolution.

### Biofluid Collection

#### Blood Sample Collection

Venous blood samples are collected in the morning after a 10–12 h fasting period from all participants according to in-house standard operating procedures. Various laboratory values (e.g., plasma cholesterol, plasma triglycerides, plasma insulin and serum high sensitivity c-reactive protein levels) are measured in a local testing laboratory [TYKSLAB, accredited according to SFS-EN ISO 15189:2013 standard by Finnish Accreditation Service (FINAS)] according to its protocols and analysis measures. All performed laboratory measurements, used methods and instruments are listed in Table 3.

**Table 3:** Laboratory measurements performed at ASIC-E4 baseline and 4-year follow-up.

	**Unit**	**Method and used instrument**
Hemoglobin	g/ L	Sysmex XN-9000
RBC count	E12/ L	Sysmex XN-9000
Hematocrit		Sysmex XN-9000
MCH	pg	Sysmex XN-9000
MCV	fL	Sysmex XN-9000
Plateles	E9/ L	Sysmex XN-9000
Total WBC count	E9/ L	Sysmex XN-9000
Neutrophils	E9/ L (%)	Sysmex XN-9000
Eosinophils	E9/ L (%)	Sysmex XN-9000
Basophils	E9/ L (%)	Sysmex XN-9000
Lymphocytes	E9/ L (%)	Sysmex XN-9000
Monocytes	E9/ L (%)	Sysmex XN-9000
Plasma insulin	mU/ L	ECLIA/ Roche/ Cobas 8000 e 602
Plasma creatinine	μmol/ L	Enzymatic/ Roche/ COBAS 8000 c 702
Serum high-sensitivity C-reactive protein	mg/ L	Immunonefelometry/ Siemens/ ProSpec
Plasma glucose	mmol/ L	Enzymatic (Hexokinase)/ Roche/ COBAS 8000 c 702
Glycated hemoglobin (HbA1c)	% (mmol/ mol)	Immunoturbidimetry/ Roche/ Cobas 6000 c 501
Plasma total cholesterol	mmol/ L	Enzymatic (CHOD-PAP)/ Roche/ cobas 8000 c 702
Plasma HDL cholesterol	mmol/ L	Enzymatic (Direct)/ Roche/ cobas 8000 c 702
Plasma LDL cholesterol	mmol/ L	Enzymatic (Direct)/ Roche/ cobas 8000 c 702
Plasma triglyserides	mmol/ L	Enzymatic (GPO-PAP)/ Roche/ cobas 8000 c 702

*HDL, High-density lipoprotein; LDL, Low-density lipoprotein; MCH, mean corpuscular hemoglobin; MCV, mean corpuscular volume; RBC, red blood cell; WBC, white blood cell*.

Additional EDTA-plasma (Vacuette EDTA-K2 tube no. 454411) and serum (Vacuette gel tube no. 454420) samples will be collected during the same session and used later for various blood biomarker measurements. Samples are gently inverted 5–10 times, centrifuged (2,200 × g, 10 min), aliquoted and stored in −80°C as 500 μl aliquots. Frozen samples will be shipped on dry ice to Clinical Neurochemistry Laboratory, Gothenburg, Sweden and stored in −80°C prior to analysis.

#### CSF Sample Collection

CSF samples will be obtained only for a subset of participants who give an additional informed consent for lumbar puncture. Refusal from the CSF sample is not an exclusion criterion for participating in the rest of the study. The CSF samples will be collected in the morning (A.M.). Fasting is not needed. The lumbar puncture will be performed under sterile conditions with the study participant either lying down or sitting, with a 20 Gauge needle. Approximately 10 ml of CSF will be tapped into a Sarstedt polypropylene tube, whereafter it will be centrifuged at 2,200 × g for 10 min, with the temperature set at +20°C. Then, 0.5–1 ml aliquots will be pipetted into cryotubes and stored at −80°C. CSF samples will be collected only once, at a time close after the participants ^11^C-UCB-J PET scan (see Figure 3 for details).

### Neuropsychological Testing

As previously described, the CERAD neuropsychological battery was completed by each participant during the screening visit, and CERAD total score > 62 was set as an inclusion criterion to the study.

In addition, an extensive neuropsychological test battery will be performed for each participant at baseline and at follow-up according to the ASIC-E4 study plan. All neuropsychological tests included in the ASIC-E4 test battery are listed in detail in Supplementary Table 1. The used tests include, e.g., the Repeatable Battery for the Assessment of Neuropsychological Status (RBANS) battery and a subset of Ravens matrices that together with CERAD orientation to time and place are used to calculate the Alzheimer's Prevention Initiative Preclinical Cognitive Composite score (APCC). The APCC is a cognitive composite score that has been shown to be sensitive to decline in preclinical AD and was used as a primary outcome measure in the API Generation Program clinical trials (45, 46).

### Data Analysis

#### Image Analysis

Pre-processing and kinetic modeling of the images will be done using Magia, an automated neuroimage analysis pipeline developed at the Human Emotion systems Laboratory, Turku PET Center (47) and in-house analysis software. Magia runs on MATLAB (The MathWorks, Inc., Natick, MA, USA), and combines methods from SPM12 (www.fil.ion.ucl.ac.uk/spm/) and FreeSurfer (https://surfer.nmr.mgh.harvard.edu/) with in-house software developed for kinetic modeling (47). Within the Magia pipeline, PET images are first motion-corrected, and co-registrated with the closest-in-time T1 MR images of the same subjects using SPM. MRI is then processed with Freesurfer for anatomical parcelation and defining both regions of interest (ROI) and the used reference region by using the Desikan-Killiany atlas (47).

^11^C-PIB uptake is quantified as standardized uptake value ratios (SUVRs) calculated 60–90 min post-injection, using cerebellar cortex as a reference region. When correlating ^11^C-PIB PET findings with other PET biomarkers, cognitive scores and fluid biomarker findings, PiB uptake will be treated as a continuous variable. However, amyloid positivity will also be defined by global ^11^C-PIB SUVR >1.5, similar to previous studies on cognitively healthy elderly populations (48, 49) and by visual read by at least two individual experts, resulting in positivity if one of the predefined cortical regions are classified as amyloid positive visually. ^11^C-PK11195 uptake is quantified as a distribution volume ratio (DVR) using a reference tissue input Logan's method within 20–60 min, where supervised clustering algorithm (SCA4) is used to calculate the clustered reference region (50). ^11^C-UCB-J uptake is quantified as distribution volume (V_T_) using a two-tissue compartment model with arterial input data. Additionally, reference tissue input modeling is done with simplified reference tissue model (SRTM) where non-displaceable binding potential (BP_ND_) is estimated with respect to centrum semiovale.

For regional analysis, ROIs presenting regions typical for Aβ deposition (pre-frontal cortex, parietal cortex, anterior cingulum, posterior cingulum, precuneus and lateral temporal cortex) as well as volume weighted amyloid composite VOI containing all the aforementioned regions as well as cerebellar cortex for reference region are created using Freesurfer and used for primary analysis. Cerebellar reference region mask created by Magia goes through two-step correction; anatomical correction removing voxels most prone to partial volume effects, and tail-exclusion removing voxels whose intensity is on the end of the radioactivity distribution (47). For ^11^C-UCB-J, a centrum semiovale ROI is drawn manually for each subject. In addition, also medial temporal cortex, and volume weighted composite VOIs for Braak I-II (entorhinal), Braak III-IV (limbic) and Braak V-VI (neocortical) will be used to investigate differences in glial activation and synaptic density in regions known to be associated with early tau deposition (51). Freesurfer regions included in each a priori amyloid and Braak VOIs are listed in Supplementary Tables 2, 3, respectively.

Cortical thickness and hippocampal volumes will be obtained from Freesurfer and adjusted for total intracranial volume (TIV) and expressed as %TIV. In addition, T1 and FLAIR sequences will be analyzed using an automatic image analysis tool (eNeuro, Combinostics Oy, Tampere, Finland), to obtain quantified total and regional white matter hyperintensities, atrophy measures, computational Fazekas scores and volumetric parameters. Lesion load, Fazekas scores and the used MRI scanner can further be used as covariates in the baseline linear models to account for vascular component and difference between the used MRI scanners.

#### Biofluid Analysis

Blood-based biomarkers for AD pathophysiology and neurodegeneration (e.g., p-tau181, p-tau231, GFAP, NfL, and soluble Aβ) will be analyzed from the blood samples using ultrasensitive Single molecule array (Simoa) technology and in-house immunoprecipitation-mass spectrometry methods both at baseline and after follow-up. Subsequently, association of these biomarkers with each other and early phase neuroimaging findings will be examined in the whole cohort and within the different *APOE* genotypes.

CSF samples will be obtained only for a subset of participants who will give an additional informed consent for lumbar punction. Due to this, number of CSF samples in the study are expected to be lower. However, we aim to measure concentrations of core CSF biomarkers for AD (total-tau, phosphorylated-tau, Aβ_1−42/1−40_) and levels of novel synaptic biomarkers from these samples.

### Statistical Plan

For descriptive statistics reported here, normally distributed data is presented as mean (SD) and non-normal data as median (interquartile range, IQR). Differences in continuous variables between the three groups were tested either with 1-way ANOVA or non-parametric Kruskal-Wallis test. Differences in categorical variables were tested using χ^2^ test. All analysis were performed using JMP Pro 16.0.0 (SAS Institute Inc.).

For subsequent data analysis in the project, analysis will include data from all study participants. If a participant will suspend the study, all data collected so far will be included. Normality of continuous variables will be inspected visually and from residuals. If required, appropriate transformations will be applied to gain normality and allow the use of parametric methods. Non-parametric methods will be applied for data that still does not follow normality assumption after transformation. If two groups are compared, student's *t*-test, Mann Whitney U-test or linear models taking account different co-variants (age, genotype, gender etc.) can be used. For three groups, 1-way ANOVA, Kruskal-Wallis test by ranks or linear models taking account different co-variants are used. For comparison of categorical variables, χ^2^ test can be used. Association between variables that are measured at one time point can be analyzed using correlations both within the whole cohort and within the three *APOE* genotypes. Pearson's correlation is applied for normally distributed data and Spearman's rank correlation for data that does not fulfill normality assumption. Association between numerical variables that will be measured more than once can be analyzed using hierarchical linear mixed models, taking into account both correlation between time points, and co-variants that affect the change.

Voxel-wise analysis for collected imaging data will be performed using SPM12 (or later) and normalized parametric images in Montreal Neurological Institute space. Results will be adjusted for multiple comparisons using false discovery rate at *P* < 0.05.

All results are considered statistically significant if *P* < 0.05 (two-sided).

## Results

Recruitment started in October 2018 and was finished in July 2020. From the 74 screened individuals, 11(14.9 %) were not eligible to the study; six individuals had contraindication to PET or MRI scan, one had CERAD total score lower than the inclusion limit of 62 points, one experienced subjective cognitive decline and three were on chronic inflammatory medication.

Sixty-three screened individuals filled all inclusion criteria and were enrolled to the following three study groups: Group 1: high risk, *APOE* ε4/ε4, N = 19; Group 2: intermediate risk, *APOE* ε4/ε3, N = 22; Group 3: typical risk, *APOE* ε3/ε3, N = 22.

### Sociodemographic Characteristics

Demographic data from the participants are presented in Table 4. A majority (61.9 %) of all participants were females and the mean age in our cohort was 67.5 (SD 4.6, range 60–75) years. The cohort includes individuals from all education levels; 30.2 % had completed primary school, 19.1 % middle or comprehensive school, 33.3% high school and 17.5 % college or university as their highest degree. The three study groups were well-matched for age (*P* = 0.91, 1-way ANOVA), sex (*P* = 0.94, χ^2^ test), education level (*P* = 0.30, χ^2^ test) and body mass index (*P* = 0.61, Kruskal-Wallis test). Approximately half (49.2 %) of all participants had a family member with AD or other memory disorder diagnosis, and the frequency was similar in all groups (*P* = 0.62, χ^2^ test). All participants were non-smokers at the time of recruitment.

**Table 4:** Baseline demographics of the ASIC-E4 study.

	**Group 1 highest risk**	**Group 2 intermediate risk**	**Group 3 typical risk**	***P***
*n*	19	22	22	
*APOE* genotype	*APOE* ε4ε4	*APOE* ε4ε3	*APOE* ε3ε3	
Age (y), mean (SD)	67.3 (4.7)	67.3 (4.8)	67.8 (4.6)	0.91
Sex (M/F), *n* (%)	7/12 (36.8/63.2)	8/14 (36.3/63.6)	9/13 (40.9/59.1)	0.94
Education, *n* (%)				0.3
Primary school	7 (36.8)	4 (18.2)	8 (36.4)	
Middle or comprehensive school	4 (21.1)	5 (22.7)	3 (13.6)	
High school	7 (36.8)	6 (27.3)	8 (36.4)	
College or university	1 (5.3)	7 (31.8)	3 (13.6)	
BMI (kg/m^2^), median (IQR)	28.4 (23.5–29.4)	26.6 (24.3–27.8)	27.0 (24.4–29.7)	0.61
Family history of AD, *n* (%)	10 (52.6)	9 (40.9)	21 (54.6)	0.62
CERAD total score, mean (SD)	84.4 (9.4)	86.0 (7.8)	85.6 (7.2)	0.80
MMSE, median (IQR)	28 (27–29)	29 (28–30)	29 (27–30)	0.053
Geriatric depression scale, median (IQR)	3.0 (0–6.0)	2.0 (0.8–3.5)	1.5 (0.3–3.8)	0.79
Medication for hypertension (*n*, %)	9 (47.4)	9 (40.9)	12 (54.6)	0.66
Medication for hyperlipidemia (*n*, %)	7 (36.8)	7 (31.8)	7 (31.8)	0.93
Antiplatelet medication (*n*, %)	8 (42.1)	3 (13.6)	7 (31.8)	0.12
Thyroid hormones (*n*, %)	5 (26.3)	4 (18.2)	2 (9.1)	0.35
Medication for asthma (*n*, %)	2 (10.5)	5 (22.7)	1 (4.6)	0.18

*AD, Alzheimer's disease; APOE, Apolipoprotein E; BMI, body mass index; CERAD, Consortium to Establish a Registry for Alzheimer's Disease; F, female; IQR, interquartile range; M, male; MMSE, mini mental state examination*.

### Clinical and Cognitive Characteristics

Clinical and cognitive characteristics of the participants are presented in Table 4. No significant differences between groups were present in mean CERAD total score (*P* = 0.80, 1-way ANOVA) or geriatric depression scale score (*P* = 0.79, Kruskal-Wallis test). The difference in MMSE score at baseline was near the level of statistical significance (*P* = 0.053, Kruskal-Wallis test), and in examining pair-wise comparisons, *APOE* ε4/ε3 heterozygotes showed higher median MMSE at baseline (29) as compared to *APOE* ε4/ε4 homozygotes (28, *P* = 0.042).

Most used medications in our cohort were for treating cardiovascular diseases; These included anti-hypertensive medication used by 47.6% (30/63), hypolipidemic drugs used by 33.3 % (21/63) and antiplatelet drugs used by 28.6 % (18/63) of all included participants. In addition, 17.5 % (11/63) used thyroid hormones and 12.7 % (8/63) had medication for asthma. Other medications used by more than one individual included low doses of anxiolytics [7.9 % (5/63)], migraine prevention [3.2 % (2/63)], treatment for insomnia [3.2% (2/63)], B12 vitamin [3.2% (3/63)] and analgesics [3.2 % (2/63)]. There were no differences between groups in any of the most frequently used medications (Table 4).

## Discussion

The ASIC-E4 study aims to investigate the early interplay of Aβ deposition, neuroinflammation and synaptic loss in cognitively unimpaired elderly with varying levels of genetic risk for AD, based on their *APOE* ε4 gene dose. We will utilize the information available at the local biobank, and the relatively high prevalence of *APOE* ε4/ε4 homozygotes in the Finnish population to recruit three groups (matched at group-level for age and sex) with different *APOE* genotype, and thus different genetic risk for future sporadic AD. Results obtained from the study will produce novel information about the gene-dose effect and possibility for early detection of AD pathological changes using various molecular imaging biomarkers, and fluid-based biomarkers more easily accessible for pharma industry and general clinical practice. The at-risk population investigated in the ASIC-E4 study carry the *APOE* ε4 risk allele for sporadic old age AD rather than the rare familial mutations causing the early-onset form of AD. Thus, the results are better generalizable at population level, especially in countries like Finland, with relatively high frequency of *APOE* ε4 carriers (approximately 32 % carry at least one ε4 allele based on the nationwide, population-based Health 2000 Health Examination Survey).

The project has many strengths that are worth mentioning. First, because of the valuable biobank collaboration, we were able to recruit also rare homozygotic carriers of the *APOE* ε4. Without this collaboration, by only relying on genotyping individual samples aiming to find the same number of such rare individuals would not be feasible. Due to the known risk effect of the *APOE* ε4 allele, most studies include *APOE* ε4 carrier status as a covariate in their analysis, or group participants as *APOE* ε4 carriers and non-carriers. However, fewer studies have investigated the gene dose effect to imaging biomarkers and compared homozygotic and heterozygotic *APOE* ε4 carriers. Aβ deposition is known to increase in a gene dose related fashion (14, 16) but less is known about its association with neuroinflammation and synaptic function *in vivo*. That said, another obvious strength of this study is the multimodal and multitracer approach that allows us to combine information from various pathophysiological routes that have been connected with AD in individuals with various levels of genetic risk for AD. Even though at this point no imaging follow-up has been planned, repeated neuropsychological testing and blood sampling allow us to investigate also effect of baseline imaging findings to future cognitive decline and blood biomarker changes.

The ASIC-E4 project does not go without limitations; first, due to delays in PET tracer production we will be unable to schedule all three PET scans within the originally planned 3-month period, but rather the ^11^C-UCB-J PET scans will be performed after ^11^C-PIB and ^11^C-PK11195. However, due to this, an amendment to the study protocol has been made; Participants gave an additional informed consent and repeated MRI scan, CERAD test battery, neuropsychological tests included in the APCC battery and gave additional blood samples. In this way, we are able to get more timely structural reference image and cognitive performance measures for the ^11^C-UCB-J PET analysis, and correct for the the possible change between baseline ^11^C-PIB/^11^C-PK11195 and ^11^C-UCB-J scans in further analysis. Secondly, even though CSF samples are included in the study protocol, they are not mandatory, and such samples are expected to be obtained from only a small subgroup of the participants. However, even though the CSF sample number will be lower and group wise comparisons most likely are not possible, they will still provide valuable way of evaluating the associations between, e.g., synaptic biomarkers in CSF with the SV2A PET imaging findings in the whole cohort. Thirdly, power analysis for the study were performed based on previously published data with the used PET tracers, thus the study might not be powered for blood biomarker comparisons between groups. Associations between the biomarker concentrations, cognitive performance and various imaging parameters can be evaluated within the whole cohort, and due to planned follow-up sample collection, longitudinal biomarker data allows us to evaluate differences in temporal change rather than solely cross-sectional differences between the groups. In addition, because the study does not include tau PET, we cannot include information on participants level of tangle pathology for our analysis.

With three PET scans, two MRI scans and multiple sampling planned for each subject, there are also risks in the project that should be evaluated and prepared for. The dropout of people during the long study protocol, especially as the global COVID-19 pandemic started during the data collection is one obvious risk for the study. To minimize this risk, changes in schedules and updates concerning the study during the pandemic have been actively communicated with the participants. So far, we have been able to continue study procedures even during the COVID-19 pandemic e.g., by providing individual transfer to the study site and private access to study facility with contacts restricted to essential study personnel following the guidelines of local authorities. In addition, to reduce the risk of dropouts, the follow-up time for cognitive testing and blood sampling was chosen to be relatively short (4 years). However, our study protocol and informed consent allows us also to contact all the study participant for possible extension of this study in the future.

To conclude, results from the ASIC-E4 study will increase the understanding of the molecular interplay between different early AD pathologies in individuals at different risk of developing AD. The used novel imaging and fluid biomarker methods will be investigated in an “at-risk” population and against cognitive changes and the results could promote the inclusion of these biomarkers into future trials investigating the effects of novel disease-modifying drugs targeted toward synaptic restoration and neuroinflammation in preclinical AD. If the hypothesized early brain changes are detected in *APOE* ε4 carriers, it could be feasible to target preventative actions toward this group in the future.

## Study Status

Study is ongoing. Recruitment of study subjects has been finished. Baseline data collection is ongoing and estimated to be completed in June 2022.

## Ethics Statement

The studies involving human participants were reviewed and approved by Ethics Committee of the Hospital District of Southwest Finland (original 17.1.2018; amendment 24.9.2018). Written informed consent was obtained from each participant both before transferring the APOE status from the biobank to the researcher, and before enrolment to this study.

## Author Contributions

JR and AS designed the study concept. AS drafted the manuscript with assistance from RL. JR and LE made major revisions to the manuscript. MP and LK contributed to the design and execution of recruitment. AS, JR, LE, NL, and MKo contributed to the design of screening, PET imaging, and sample collection. AS, JT, VO, and RA contributed to PET imaging, modelling, and analysis protocols. VS, JS, and RP contributed to the design of MRI data collection. EL contributed to the design of statistical analysis plan. MKa contributed to the design of the used neuropsychological test battery. HZ and KB contributed to the planning of sample collection and fluid biomarker analysis. All authors contributed to the conception and design of different parts of the study protocol, made revisions to the manuscript, and approved the submitted version.

## Funding

AS has receive funding for this study from Finnish Governmental Research Funding (ERVA) for Turku University Hospital, Emil Aaltonen Foundation, Orion Research Foundation sr, Paulo Foundation and Academy of Finland (#341059). LE has received funding from the Emil Aaltonen Foundation. HZ is a Wallenberg Scholar supported by grants from the Swedish Research Council (#2018-02532), the European Research Council (#681712), Swedish State Support for Clinical Research (#ALFGBG-720931), the Alzheimer Drug Discovery Foundation (ADDF), USA (#201809-2016862), the AD Strategic Fund and the Alzheimer's Association (#ADSF-21-831376-C, #ADSF-21-831381-C, and #ADSF-21-831377-C), the Olav Thon Foundation, the Erling-Persson Family Foundation, Stiftelsen för Gamla Tjänarinnor, Hjärnfonden, Sweden (#FO2019-0228), the European Union's Horizon 2020 research and innovation programme under the Marie Skłodowska-Curie grant agreement No. 860197 (MIRIADE), and the UK Dementia Research Institute at UCL. KB is supported by the Swedish Research Council (#2017-00915), the Alzheimer Drug Discovery Foundation (ADDF), USA (#RDAPB-201809-2016615), the Swedish Alzheimer Foundation (#AF-742881), Hjärnfonden, Sweden (#FO2017-0243), the Swedish state under the agreement between the Swedish government and the County Councils, the ALF-agreement (#ALFGBG-715986), the European Union Joint Program for Neurodegenerative Disorders (JPND2019-466-236), the National Institute of Health (NIH), USA, (grant #1R01AG068398-01), and the Alzheimer's Association 2021 Zenith Award (ZEN-21-848495). JR has received funding from the Academy of Finland (#310962), Sigrid Juselius Foundation and Finnish Governmental Research Funding (VTR) for Turku University Hospital. Funds for open access publication fees were received from Turku University Hospital Research Services.

## Conflict of Interest

HZ has served at scientific advisory boards and/or as a consultant for Abbvie, Alector, Annexon, Artery Therapeutics, AZTherapies, CogRx, Denali, Eisai, Nervgen, Pinteon Therapeutics, Red Abbey Labs, Passage Bio, Roche, Samumed, Siemens Healthineers, Triplet Therapeutics, and Wave, has given lectures in symposia sponsored by Cellectricon, Fujirebio, Alzecure and Biogen, and is a co-founder of Brain Biomarker Solutions in Gothenburg AB (BBS), which is a part of the GU Ventures Incubator Program. KB has served as a consultant, at advisory boards, or at data monitoring committees for Abcam, Axon, Biogen, JOMDD/Shimadzu. Julius Clinical, Lilly, MagQu, Novartis, Pharmatrophix, Prothena, Roche Diagnostics, and Siemens Healthineers, and is a co-founder of Brain Biomarker Solutions in Gothenburg AB (BBS), which is a part of the GU Ventures Incubator Program. The remaining authors declare that the research was conducted in the absence of any commercial or financial relationships that could be construed as a potential conflict of interest.

## Publisher's Note

All claims expressed in this article are solely those of the authors and do not necessarily represent those of their affiliated organizations, or those of the publisher, the editors and the reviewers. Any product that may be evaluated in this article, or claim that may be made by its manufacturer, is not guaranteed or endorsed by the publisher.
