# Supplementary material for: ASIC-E4: Interplay of Beta-Amyloid, Synaptic Density and Neuroinflammation in Cognitively Normal Volunteers With Three Levels of Genetic Risk for Late-Onset Alzheimer's Disease – Study Protocol and Baseline Characteristics
Source: Front Neurol. 2022 Feb 9;13:826423. doi: 10.3389/fneur.2022.826423 (PMC8863967; doi:10.3389/fneur.2022.826423)
Supplement: Supplementary file 1 [file Data_Sheet_1.pdf]

Supplementary material

Study protocol

ASIC-E4: Interplay of beta-amyloid, synaptic density and neuroinflammation in cognitively normal volunteers with three levels of genetic risk for late-onset Alzheimer's disease – Study protocol and baseline characteristics

Supplementary Table 1 Cognitive test batteries included in screening, baseline and follow-up visits of the ASIC-E4 study

CERAD test battery was used for screening of participants, and other listed cognitive test batteries and subtests were part of the ASIC-E4 test battery used at baseline and at 4-year follow up. Due to a delay in the availability of ^{11}C-UCB-J, CERAD and RBANS test batteries and the used subset of Raven's matrices were performed also at the time of ^{11}C-UCB-J scans.

<table>
<tr><th>Test battery</th><th>Subtest</th><th>Cognitive domain</th><th>Screening</th><th>Baseline (PIB/PK)</th><th>Baseline (UCB-J)</th><th>4y Follow-up</th></tr>
<tr><td>CERAD test battery</td><td>Full CERAD battery</td><td>Global cognition</td><td>x</td><td>n/a</td><td>x</td><td>x</td></tr>
<tr><td>Trail-making A and B</td><td></td><td>Executive function</td><td>n/a</td><td>x</td><td>n/a</td><td>x</td></tr>
<tr><td>Stroop test</td><td></td><td>Executive function</td><td>n/a</td><td>x</td><td>n/a</td><td>x</td></tr>
<tr><td>S-fluency</td><td></td><td>Verbal fluency</td><td>n/a</td><td>x</td><td>n/a</td><td>x</td></tr>
<tr><td rowspan="6">WMS-R</td><td>Logical memory I</td><td rowspan="3">Immediate memory</td><td rowspan="6">n/a</td><td rowspan="6">x</td><td rowspan="6">n/a</td><td rowspan="6">x</td></tr>
<tr><td>Verbal learning I</td></tr>
<tr><td>Visual reproduction I</td></tr>
<tr><td>Logical memory II</td><td rowspan="3">Delayed memory</td></tr>
<tr><td>Verbal learning II</td></tr>
<tr><td>Visual reproduction II</td></tr>
<tr><td rowspan="4">WAIS-R</td><td>Subtest 6, Block design</td><td>Perceptual reasoning</td><td rowspan="4">n/a</td><td rowspan="4">x</td><td rowspan="4">n/a</td><td rowspan="4">x</td></tr>
<tr><td>Subtest 10, Coding</td><td>Cognitive processing speed</td></tr>
<tr><td>Subtest 11, Similarities</td><td>Verbal comprehension</td></tr>
<tr><td>Subtest 7, Digit span</td><td>Working memory</td></tr>
<tr><td>FCSRT</td><td></td><td>Episodic memory</td><td>n/a</td><td>x</td><td>n/a</td><td>x</td></tr>
<tr><td>Boston naming test</td><td></td><td>Language</td><td>n/a</td><td>x</td><td>n/a</td><td>x</td></tr>
<tr><td rowspan="4">RBANS</td><td>Full immediate memory index</td><td>Immediate memory</td><td rowspan="4">n/a</td><td rowspan="4">x</td><td rowspan="4">x</td><td rowspan="4">x</td></tr>
<tr><td>Full visuospatial/constructional index</td><td>Visuospatial/constructional skills</td></tr>
<tr><td>Full language index</td><td>Language</td></tr>
<tr><td>Full attention index</td><td>Attention</td></tr>
</table>

	Full delayed memory index	Delayed memory				
	Sum of index scores					
	RBANS total scale index score		*n/a*	**x**	**x**	**x**
**APCC**	MMSE, Orientation to time	Orientation	*n/a*	**x**	**x**	**x**
	MMSE, Orientation to place		*n/a*	**x**	**x**	**x**
	A subset of Ravens matrices (A2, A4, A8, B1-B6)	Visuospatial skills	*n/a*	**x**	**x**	**x**
	RBANS, Line orientation		*n/a*	**x**	**x**	**x**
	RBANS, Coding	Attention	*n/a*	**x**	**x**	**x**
	RBANS, List recall	Episodic memory	*n/a*	**x**	**x**	**x**
	RBANS, Story recall	Episodic memory	*n/a*	**x**	**x**	**x**
	APCC score		*n/a*	**x**	**x**	**x**
**PACC5**	FCRST, Total recall	Episodic memory	*n/a*	**x**	*n/a*	**x**
	WMS-R; Logical memory II	Delayed memory	*n/a*	**x**	*n/a*	**x**
	WAIS-R; Subtest 10, Coding	Cognitive processing speed	*n/a*	**x**	*n/a*	**x**
	MMSE	Global cognition	*n/a*	**x**	**x**	**x**
	CERAD; Verbal fluency	Language	*n/a*	**x**	**x**	**x**
	PACC5 score		*n/a*	**x**	*n/a*	**x**

Abbreviations: *n/a*; Not applicable; *APCC*, Alzheimer's Prevention Initiative Composite Cognitive Test Score; *CERAD*, Consortium to Establish a Registry for Alzheimer's Disease; *FCSRT*, The Free and Cued Selective Reminding Test; *MMSE*, Mini Mental State Examination; *PACC5*, Preclinical Alzheimer's disease Cognitive Composite 5; *RBANS*, Repeatable Battery for the Assessment of Neuropsychological Status; *WAIS-R*, Wechsler Adult Intelligence Scale—Revised; *WMS-R*, Wechsler Memory Scale-Revised.

Supplementary Table 2 **Freesurfer regions used to define the pre-defined volumes-of-interest (VOIs) for beta-amyloid deposition related regions.**

Freesurfer label name	Freesurfer label #	VOI name
ctx-lh-caudalmiddlefrontal	1003	Prefrontal cortex
ctx-lh-lateralorbitofrontal	1012	Prefrontal cortex
ctx-lh-medialorbitofrontal	1014	Prefrontal cortex
ctx-lh-parsopercularis	1018	Prefrontal cortex
ctx-lh-parsorbitalis	1019	Prefrontal cortex
ctx-lh-parstriangularis	1020	Prefrontal cortex
ctx-lh-rostralmiddlefrontal	1027	Prefrontal cortex
ctx-lh-frontalpole	1032	Prefrontal cortex
ctx-rh-caudalmiddlefrontal	2003	Prefrontal cortex
ctx-rh-lateralorbitofrontal	2012	Prefrontal cortex
ctx-rh-medialorbitofrontal	2014	Prefrontal cortex
ctx-rh-parsopercularis	2018	Prefrontal cortex
ctx-rh-parsorbitalis	2019	Prefrontal cortex
ctx-rh-parstriangularis	2020	Prefrontal cortex
ctx-rh-rostralmiddlefrontal	2027	Prefrontal cortex
ctx-rh-frontalpole	2032	Prefrontal cortex
ctx-lh-inferiorparietal	1008	Parietal cortex
ctx-lh-superiorparietal	1029	Parietal cortex
ctx-lh-supramarginal	1031	Parietal cortex
ctx-rh-inferiorparietal	2008	Parietal cortex
ctx-rh-superiorparietal	2029	Parietal cortex
ctx-rh-supramarginal	2031	Parietal cortex
ctx-lh-inferiortemporal	1009	Lateral temporal cortex
ctx-lh-middletemporal	1015	Lateral temporal cortex
ctx-lh-superiortemporal	1030	Lateral temporal cortex
ctx-lh-temporalpole	1033	Lateral temporal cortex
ctx-lh-transversetemporal	1034	Lateral temporal cortex
ctx-rh-inferiortemporal	2009	Lateral temporal cortex

ctx-rh-middletemporal	2015	Lateral temporal cortex
ctx-rh-superiortemporal	2030	Lateral temporal cortex
ctx-rh-temporalpole	2033	Lateral temporal cortex
ctx-rh-transversetemporal	2034	Lateral temporal cortex
ctx-lh-lateraloccipital	1011	Lateral temporal cortex
ctx-rh-lateraloccipital	2011	Lateral temporal cortex
ctx-lh-precuneus	1025	Precuneus
ctx-rh-precuneus	2025	Precuneus
ctx-lh-caudalanteriorcingulate	1002	Anterior cingulum
ctx-lh-rostralanteriorcingulate	1026	Anterior cingulum
ctx-rh-caudalanteriorcingulate	2002	Anterior cingulum
ctx-rh-rostralanteriorcingulate	2026	Anterior cingulum
ctx-lh-isthmuscingulate	1010	Posterior cingulum
ctx-lh-posteriorcingulate	1023	Posterior cingulum
ctx-rh-isthmuscingulate	2010	Posterior cingulum
ctx-rh-posteriorcingulate	2023	Posterior cingulum
Left-Cerebellum-Cortex	8	Cerebellar cortex
Right-Cerebellum-Cortex	47	Cerebellar cortex
Left-Hippocampus	17	Medial temporal cortex
Left-Amygdala	18	Medial temporal cortex
ctx-lh-entorhinal	1006	Medial temporal cortex
Right-Hippocampus	53	Medial temporal cortex
Right-Amygdala	54	Medial temporal cortex
ctx-rh-entorhinal	2006	Medial temporal cortex

Suplementary Table 3 **Freesurfer regions used to define the Braak volumes-of-interest (VOIs) for tau deposition related regions.**

Freesurfer label name	Freesurfer label #	VOI name
L Entorhinal cortex	1006	Braak I
R Entorhinal cortex	2006	Braak I
L Hippocampus	17	Braak II
R Hippocampus	53	Braak II
L Parahippocampal gyrus	1016	Braak III
L Fusiform	1007	Braak III
L Lingual gyrus	1013	Braak III
L Amygdala	18	Braak III
R Parahippocampal gyrus	2016	Braak III
R Fusiform	2007	Braak III
R Lingual gyrus	2013	Braak III
R Amygdala	54	Braak III
L Middle temporal ctx	1015	Braak IV
L Thalamus	10	Braak IV
L CaudAnt Cingulate	1002	Braak IV
L RostAnt Cingulate	1026	Braak IV
L Post Cingulate	1023	Braak IV
L Isthmus Cingulate	1010	Braak IV
L Insula	1035	Braak IV
L Inferior temporal ctx	1009	Braak IV
L Temporal Pole	1033	Braak IV
R Middle temporal ctx	2015	Braak IV
R Thalamus	49	Braak IV
R CaudAnt Cingulate	2002	Braak IV
R RostAnt Cingulate	2026	Braak IV
R Post Cingulate	2023	Braak IV
R Isthmus Cingulate	2010	Braak IV
R Insula	2035	Braak IV
R Inferior temporal ctx	2009	Braak IV

R Temporal Pole	2033	Braak IV
L Frontal SUPFR	1028	Braak V
L Frontal FPORB	1012	Braak V
L Frontal FPORB	1014	Braak V
L Frontal FPORB	1032	Braak V
L Frontal MIDFR	1003	Braak V
L Frontal MIDFR	1027	Braak V
L Frontal PARSFR	1018	Braak V
L Frontal PARSFR	1019	Braak V
L Frontal PARSFR	1020	Braak V
L Caudate	11	Braak V
L Putamen	12	Braak V
L Lateral Occipital ctx	1011	Braak V
L Parietal Supramarginal ctx	1031	Braak V
L Parietal Inferior ctx	1008	Braak V
L Superior Temporal ctx	1030	Braak V
L Pallidum	13	Braak V
L Parietal Superior	1029	Braak V
L Precuneus	1025	Braak V
L Banks of superior temporal sulcus	1001	Braak V
L Nucleus accumbens	26	Braak V
L Transverse Temporal ctx	1034	Braak V
R Frontal SUPFR	2028	Braak V
R Frontal FPORB	2012	Braak V
R Frontal FPORB	2014	Braak V
R Frontal FPORB	2032	Braak V
R Frontal MIDFR	2003	Braak V
R Frontal MIDFR	2027	Braak V
R Frontal PARSFR	2018	Braak V
R Frontal PARSFR	2019	Braak V
R Frontal PARSFR	2020	Braak V
R Caudate	50	Braak V

R Putamen	51	Braak V
R Lateral Occipital ctx	2011	Braak V
R Parietal Supramarginal ctx	2031	Braak V
R Parietal Inferior ctx	2008	Braak V
R Superior Temporal ctx	2030	Braak V
R Pallidum	52	Braak V
R Parietal Superior ctx	2029	Braak V
R Precuneus	2025	Braak V
R Banks of superior temporal sulcus	2001	Braak V
R Nucleus accumbens	58	Braak V
R Transverse Temporal ctx	2034	Braak V
L Pericalcarine	1021	Braak VI
L Postcentral gyrus	1022	Braak VI
L Cuneus	1005	Braak VI
L Precentral gyrus	1024	Braak VI
L Paracentral gyrus	1017	Braak VI
R Pericalcarine	2021	Braak VI
R postcentral gyrus	2022	Braak VI
R Cuneus	2005	Braak VI
R Precentral gyrus	2024	Braak VI
R Paracentral gyrus	2017	Braak VI
